# Epitope Mapping of Metuximab on CD147 Using Phage Display and Molecular Docking

**DOI:** 10.1155/2013/983829

**Published:** 2013-06-03

**Authors:** Bifang He, Canquan Mao, Beibei Ru, Hesong Han, Peng Zhou, Jian Huang

**Affiliations:** ^1^Center of Bioinformatics (COBI), Key Laboratory for NeuroInformation of Ministry of Education, University of Electronic Science and Technology of China, Chengdu 610054, China; ^2^School of Life Science and Engineering, Southwest Jiaotong University, Chengdu 610031, China

## Abstract

Metuximab is the generic name of Licartin, a new drug for radioimmunotherapy of hepatocellular carcinoma. Although it is known to be a mouse monoclonal antibody against CD147, the complete epitope mediating the binding of metuximab to CD147 remains unknown. We panned the Ph.D.-12 phage display peptide library against metuximab and got six mimotopes. The following bioinformatics analysis based on mimotopes suggested that metuximab recognizes a conformational epitope composed of more than 20 residues. The residues of its epitope may include T28, V30, K36, L38, K57, F74, D77, S78, D79, D80, Q81, G83, S86, N98, Q100, L101, H102, G103, P104, V131, P132, and K191. The homology modeling of metuximab and the docking of CD147 to metuximab were also performed. Based on the top one docking model, the epitope was predicted to contain 28 residues: AGTVFTTV (23–30), I37, D45, E84, V88, EPMGTANIQLH (92–102), VPP (131–133), Q164, and K191. Almost half of the residues predicted on the basis of mimotope analysis also appear in the docking result, indicating that both results are reliable. As the predicted epitopes of metuximab largely overlap with interfaces of CD147-CD147 interactions, a structural mechanism of metuximab is proposed as blocking the formation of CD147 dimer.

## 1. Introduction

Metuximab is the generic name of HAb18, a mouse monoclonal antibody of IgG1 class developed by Chen et al. in 1989 [1]. The hybridoma producing HAb18 was made from mice immunized with a cell suspension of fresh human hepatocellular carcinoma tissues. The antigen recognized by HAb18 was accordingly called HAb18G, which was later identified as an isoform of basigin [2]. The products of basigin gene have many well-known names, for example, cluster of differentiation 147 (CD147), extracellular matrix metalloproteinase inducer (EMMPRIN), and so on. In this paper, we use CD147 hereafter to refer to the antigen recognized by metuximab.

CD147 is a transmembrane glycoprotein of the immunoglobulin superfamily. It has been involved in various physiological functions such as spermatogenesis [3], embryo implantation [4], tissue remodeling [5], and diverse pathological processes such as neuroinflammation [6], Alzheimer's disease [7], malaria infection [8], and tumor progression [9]. Though widely expressed on numerous cell types, CD147 is highly enriched on the surface of cancer cells, especially on those of epithelial origin, for example, breast cancer [10] and liver cancer [11]. Thus, it has been taken as a biomarker that can be used in cancer detection [12]. Furthermore, CD147 has also been proposed to be a new drug target for developing therapeutics against inflammation, malaria [13], and cancer [14]. Metuximab is a success case which targets CD147. The iodine-131-labeled F(ab′)2 fragment of metuximab has been reported to be safe and effective for targeted treatment of hepatocellular carcinoma in clinical trials [15, 16]. The injection with the brand name Licartin was approved as a new drug for radioimmunotherapy of hepatocellular carcinoma by the State Food and Drug Administration, China, in 2005. 

Where does metuximab bind to CD147? The answer will help us understand the mechanism of CD147 function and benefit the development of new drugs targeting CD147. Using binding assays to a series of truncated fragments of CD147 ectodomain, Ku et al. reported that the segment of 39LTCSLNDSATEV50 was the epitope on CD147 recognized by metuximab [17]. Yu et al. docked the Fv fragment of metuximab onto the N-terminal domain of CD147 in a head-to-head manner when the crystal structure of CD147 ectodomain was resolved [18]. Their model and experiment results suggested the residues E49, T51, and D65 on CD147 might also play an important role in the interaction between CD147 and metuximab [18]. However, a panorama of the epitope mediating the binding of metuximab to CD147 has not been proposed yet.

Crystallographic analysis of antigen-antibody complex is the most accurate approach to mapping an epitope. However, it is time consuming and sometimes technically difficult or even impossible to get an antigen-antibody complex crystallized. As an alternative choice for epitope mapping with lower but acceptable precision, mimotope analysis is becoming to be an increasingly popular method for its cheapness and quickness [19, 20]. In this study, the epitope of metuximab was defined completely at the residue level using phage display and the following bioinformatics analysis. The result was then validated using molecular modeling and docking. The panoramic model where CD147 is recognized by metuximab will provide valuable information and better structural basis for decoding CD147 and developing relevant drugs.

## 2. Materials and Methods

### 2.1. Biopanning of a 12-Mer Phage Display Library

The F(ab′)2 fragment of metuximab as freeze-dried powder with purity above 97% was provided by Chengdu Huasun Bio-Tech Co. LTD. The Ph.D.-12 phage display peptide library that displays 2.7 × 10^9^ unique 12-amino acid peptides fused to the pIII minor coat protein of the M13 filamentous phage was purchased from New England BioLabs. Three successive rounds of biopannings were performed with the F(ab′)2 fragment of metuximab as the capture reagent coated on 96-well microtiter plates, as described in the manufacturer's manual with modifications. 

In brief, the sample was diluted to a concentration of 200 *μ*g/mL in 0.1 M NaHCO3 (pH 8.6). 100 *μ*L of the above solution was transferred to a 96-well plate and incubated overnight at 4°C with gentle agitation in a humidified container. Wells coated with buffer were used as the negative control. The coating solution was removed from the wells, and the plate was tapped onto a clean paper towel to remove residual solution. Each well was filled with the blocking buffer (0.1 M sodium bicarbonate buffer pH 8.6 plus 5 mg/mL BSA). The plate was incubated for 1 hour at 4°C. The blocking solution was removed, and the plate was tapped onto a clean paper towel to remove residual solution. Each well was washed rapidly 6 times with 1% TBST (Tris-buffered saline with 0.1% Tween 20). The plate was swirled repeatedly when coating and washing to ensure that each well including its sides was coated and washed completely. 2 × 10^11^ phages from the library were mixed with 100 *μ*L TBST and transferred onto coated well. The plate was incubated at room temperature for 45 min with gentle rocking. Then, the supernatant was removed and the plate was washed ten times with 200 *μ*L TBST buffer. The bound phages were eluted with 100 *μ*L 0.2 M glycine-HCl (pH 2.2) plus 1 mg/mL of BSA. The eluate was transferred to an Eppendorf tube and neutralized immediately by adding 30 *μ*L 1 M Tris-HCl (PH9.1). After titration, the eluate was amplified in *E. coli* strain ER2738 culture for additional two pannings using the same PFUs of total phage in each round. After the third panning experiment, the final eluate was mixed with ER2738 host cells, diluted, and spread on LB-Xgal/IPTG plates. Twenty isolated plaques were randomly picked and amplified for DNA sequencing.

### 2.2. Epitope Mapping Based on Mimotope Analysis

The peptides displayed on the selected phages were deduced from the results of DNA sequencing. The data was firstly cleaned using the tools in the SAROTUP suite to exclude any possible target-unrelated peptides [21–23]. The left peptides were then mapped back to the surface of CD147 based on its crystal structure (PDB: 3B5H) using the EpiSearch program by default parameters [24]. The mapping result of each peptide was united to make the epitope on CD147 recognized by metuximab. 

### 2.3. Molecular Modeling and Docking

The sequences of the variable heavy chain (VH) and light chain (VL) of metuximab were extracted from the United State patent with the number US7638619 [25]. The corresponding GenBank accession numbers of VH and VL are ADC21949.1 and ADC21950.1, respectively. The sequences were manually checked. Only segments 20–136 of VH and 21–130 of VL were submitted to the RosettaAntibody server to construct the model for variable domain (Fv) of metuximab [26]. For each framework region and complementarity determining region (CDR) of metuximab, the best templates were used for homology modeling (see Table 1).

**Figure 1:**
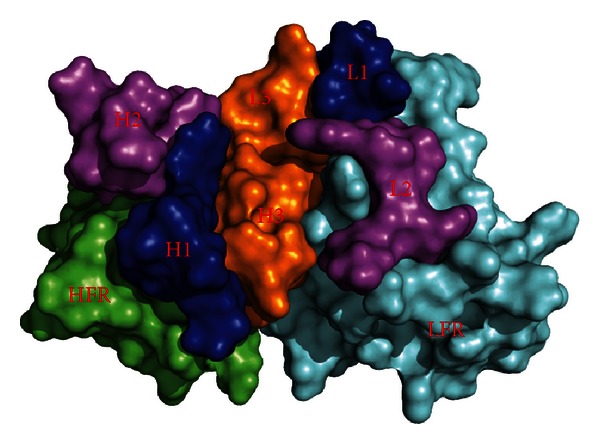
Fv model of metuximab.

As shown in Table 1, the H3 loop of metuximab does not have any sequence match. It was thus modeled ab initio by the RosettaAntibody server [26]. Top ten models with energy minimized were given by the server, and the top one (see Figure 1) was used as the receptor for docking. 

The 3D structure of CD147 monomer (3B5H, chain C) was then docked to the Fv model of metuximab using the program ZDOCK with framework region blocked [27]. The results were evaluated using ZRANK and optimized using RDOCK [28, 29]. The top one docking model from RDOCK was picked up as the theoretical metuximab-CD147 complex. The interfaces between metuximab and CD147 were then computed with the program PISA [30]. The one on CD147 side was taken as the epitope produced by molecular docking. 

## 3. Results and Discussion

### 3.1. Analysis on Panning Results

The titer of the eluate after each round of pannings increased from 10^3^, 10^4^ to 10^6^ PFU/mL, indicating an efficient enrichment of phages specifically binding to metuximab. After the third panning, twenty phage clones were randomly picked and sent to DNA sequencing. The deduced amino acids sequences are listed in Table 2.

As shown in Table 2, seven unique sequences were obtained from biopannings. Among them, the peptide YPHFHKHTLRGH is most frequent. Just by visual inspection, these peptides can be grouped into 4 clusters, that is, P1 and P2, P3 and P4, P5 and P6, and P7. 

### 3.2. Epitope Mapping Results from Mimotope Analysis

These peptides were checked using tools in the SAROTUP suite. Interestingly, it was reported that the peptide DFDVSFLSARMR had also been panned out from the Ph.D.-12 phage display peptide library using the protein tonB of *E. coli* [31]. To avoid any possible TUP, this peptide was dropped from the following epitope mapping based on mimotopes. The left peptides were then used together as inputs of the EpiSearch program [24]. By default parameters, they were mapped back to the surface of CD147 monomer based on the crystal structure (PDB: 3B5H, Chain C). The analysis found two solutions centered at residue Lys191 (score = 0.780) and Leu101 (score = 0.713), respectively. For each solution, the high-scoring patch for each peptide was united to make the whole epitope predicted to be recognized by metuximab. 

**Figure 2:**
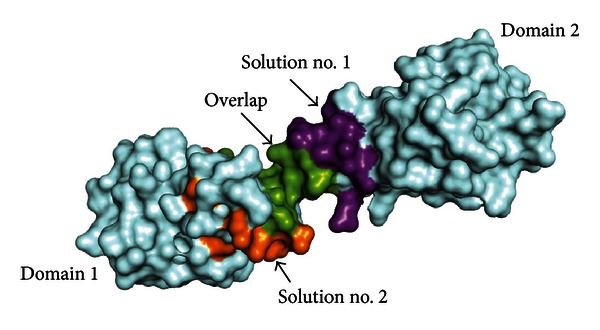
Epitope mapping results from mimotope analysis. Residues only appearing in solutions nos. 1 and 2 are colored in purple and orange, respectively; the overlapping region of the two solutions is drawn in green. All other parts of CD147 surface are presented in pale cyan.

As shown in Tables 3 and 4, the two epitopes predicted by EpiSearch are similar in size and residue composition as well. For example, both of them contain 22 residues in total. Furthermore, 11 residues, printed in italics in Tables 3 and 4, are identical in two solutions, indicating a consistence in some degree between the two solutions. As shown in Figure 2, most residues of solution no. 1 locate on the domain 2 of CD147 and the main part of solution no. 2 is on the surface of the domain 1. The two solutions overlap at the area between the two domains of CD147. 

When the parameter “Accuracy cutoff” (allowed mismatch) was set from 3 (default) to 2 (a stricter value), only solution 2 was left. Thus, solution 2 might be a more accurate prediction, and we considered this solution to be the epitope predicted by phage display and mimotope analysis.

It has been reported that the frequencies of peptides are not correlated to their binding strength and the diversity of peptides are important in analysis [32]. Therefore, all peptides were treated equally in our study, although their occurrences in the panning results were quite different. Indeed, the P1 mimotope YPHFHKHTLRGH, the most frequently appeared peptide, did not contain more epitope residues than others when all these peptides were mapped back to the surface of CD147 and compared with the docking results.

We have also used other tools such as PepMapper [33–35] to interpret the phage display data and got some results similar to EpiSearch. This makes the prediction above even convincing. 

### 3.3. Epitope Mapping Results from Molecular Docking

**Figure 3:**
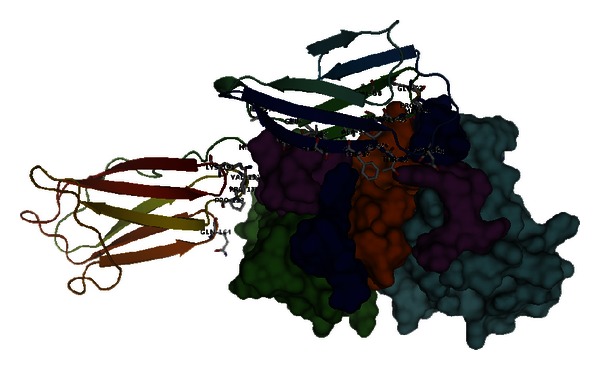
Theoretical model of metuximab-CD147 complex. CD147 was displayed as cartoon in rainbow color, and the interface residues on it were drawn as sticks and labeled.

The computation of the top one model from RDOCK results revealed that metuximab might bind to a conformational epitope. As shown in Figure 3, the epitope was predicted to contain 28 residues: 23AGTVF**T**T**V**30, I37, D45, E84, V88, 92EPMGTA**N**I**QLH**102, 131**VP**P133, Q164, and **K**191.

Other nine models of the top ten RDOCK poses were also used to compute the theoretical epitopes. The results show that seven of them were identical to that of top one model. The epitope derived from the 7th model is a little bit different. Besides including all residues shown in Figure 3, it also contains residues N44, T48, and F89. Only the epitope derived from the 5th model is quite different from that of the top one model. Since the computational results of top ten models are quite consistent with top one, we believe the epitope shown in Figure 3 is reasonable. 

Though there are arguments about the role of molecular docking in epitope mapping, the results of molecular docking can be very accurate. In 2001, Saphire et al. solved the structure of b12, a neutralizing human IgG against HIV-1. They docked gp120 to b12 and predicted the epitope recognized by b12 [36]. Six years later, the crystal structure of gp120 and b12 complex was solved and the true epitope was found to be nearly the same as the prediction [37]. As the structure of metuximab-CD147 complex is not solved yet, the accuracy of our docking results cannot be validated at present. However, when the results of mimotope analysis and molecular docking were compared, a significant overlap was found. For example, almost half residues of the phage display solution no. 2 also appear in the docking result (see the residues in bold in Section 3.3). The consistency between phage display and molecular docking suggests that both predictions are reliable. 

### 3.4. Structural Insights into Metuximab Mechanism

CD147 exists in several forms, such as monomer, dimer, and polymer [18]. It has been observed that CD147 can bind to soluble CD147 [38]. Very recently, Cui et al. found that dimerization was essential for CD147 to promote tumor invasion via MAPK pathway [39]. According to the solved crystal structure of CD147 (3B5H), the interactions between the four monomer chains (i.e., chain A, B, C, and D) of CD147 might represent four possible ways to form a CD147 dimer [18]. Two CD147 monomers on the membranes of two cells may interact with each other via their N-terminal domain (domain 1) just like forming the AC or BC dimer. A soluble CD147 may also bind to another CD147 on cell membrane through its C-terminal domain (domain 2), which is similar to AD dimer. The interaction between chain D and D′ is deemed a result from crystal packing, which is mainly mediated by 59GVVKEDA66 [18].

Very interestingly, the predicted epitopes of metuximab based on either phage display or molecular docking overlap the interfaces between AC, BC, and AD dimmers. Coincidentally, the nonspecific D D′ dimer is the only exception, which has no intersection with the epitope of metuximab. Thus, at least one mechanism of metuximab is to block CD147-CD147 interactions. Therefore, peptides obtained from screening the phage-displayed random peptide library might also have potential applications in blocking CD147 pathways. 

## 4. Conclusions

According to the results from and analyses on molecular docking and phage display experiments, we conclude that metuximab recognizes a conformational epitope composed of more than 20 residues. These residues mainly locate on the N-terminal domain surface of CD147 and largely overlap with interfaces of CD147-CD147 interactions. Blocking the formation of CD147-CD147 dimer may be an important mechanism of metuximab function. 

## Figures and Tables

**Table 1:** Templates used in modeling metuximab.

Region*	PDB	Identity	Align length	Mismatches	Gap openings	*E* value
HFR	1SBS	95.52	67	3	0	0.00*E* + 00
LFR	1IAI	85.48	62	9	0	3.00*E* − 22
H1	2DLF	100	10	0	0	1.00*E* − 07
H2	2DLF	75	20	3	2	3.00*E* − 08
H3	1A0Q	0	0	0	0	0.00*E* + 00
L1	1QNZ	100	6	0	0	5.80*E* − 02
L2	3FCT	83.33	6	1	0	3.30*E* − 01
L3	1NCD	77.78	9	2	0	5.00*E* − 03

*HFR: heavy chain framework region; LFR: light chain framework region; H1, H2, H3, L1, L2, and L3 refer to CDR1, CDR2, and CDR3 loops of heavy and light chain, respectively.

**Table 2:** Peptides selected from Ph.D.-12 phage display peptide library using metuximab.

Number	Sequence	Occurrence
P1	Y PHFHKHTLRGH	9
P2	YPHFHKHSLRGQ	1
P3	DHKPFKPTHRTL	1
P4	FHKPFKPTHRTL	1
P5	QSSCHKHSVRGR	1
P6	QSSFSNHSVRRR	1
P7	DFDVSFLSARMR	6

**Table 3:** Epitope mapping solution no. 1 based on mimotope analysis.

Mimotope	Predicted epitopic residues
Y PHFHKHTLRGH	L101, H102, G103, P104, R106, P132, P133, T135, T188, K191, G192
YPHFHKHSLRGQ	S78, Q81, Q100, L101, H102, G103, P104, R106, S128, S130, P132, P133, S189, S190, K191, G192, S193
DHKPFKPTHRTL	L101, H102, P104, R106, P132, P133, T135, T188, K191, D194
FHKPFKPTHRTL	L101, H102, P104, R106, P132, P133, T135, T188, K191
QSSCHKHSVRGR	V30, S78, Q81, Q100, H102, G103, R106, S128, S130, V131, S189, S190, K191, G192, S193
QSSFSNHSVRRR	V30, S78, Q81, Q100, H102, R106, S128, S130, V131, S189, S190, S193

Union	*V30*, *S78, Q81, Q100, L101, H102, G103, P104*, **R106**, **S128**, **S130**, *V131*, *P132*, **P133**, **T135**, **T188**, **S189**, **S190**, *K191*, **G192**, **S193**, **D194**; 22 residues in total.

**Table 4:** Epitope mapping solution no. 2 based on mimotope analysis.

Mimotope	Predicted epitopic residues
Y PHFHKHTLRGH	T28, K36, L38, K57, F74, G83, L101, H102, G103, P104, P132, K191
YPHFHKHSLRGQ	K36, L38, K57, F74, S78, Q81, G83, S86, Q100, L101, H102, G103, P104, P132, K191
DHKPFKPTHRTL	T28, K36, L38, K57, F74, D77, D79, D80, L101, H102, P104, P132, K191
FHKPFKPTHRTL	T28, K36, L38, K57, F74, L101, H102, P104, P132, K191
QSSCHKHSVRGR	V30, K36, K57, S78, Q81, G83, S86, Q100, H102, G103, V131, K191
QSSFSNHSVRRR	V30, F74, S78, Q81, S86, N98, Q100, H102, V131

Union	**T28**, *V30*, **K36**, **L38**, **K57**, **F74**, **D77**, *S78*, **D79**, **D80**, *Q81*, **G83**, **S86**, **N98**, *Q100*, *L101*, *H102*, *G103*, *P104*, *V131*, *P132*, *K191*; 22 residues in total.

## References

[1] Chen ZN, Liu YF, Yang JZ (1989). Production of a monoclonal antibody against human hepatocellular carcinoma and locating the corresponding antigen P60 using immunohistochemistry. *Dan Ke Long Kang Ti Tong Xun*.

[2] Chen ZN, Yang Z, Mi L, Jiang JL, Guo X (1999). Analysis on the structure and function of hepatoma transfer-associated factor HAb18G. *Journal of Cellular and Molecular Immunology*.

[3] Chen H, Lam Fok K, Jiang X, Chan HC (2012). New insights into germ cell migration and survival/apoptosis in spermatogenesis: lessons from CD147. *Spermatogenesis*.

[4] Chen L, Belton RJ, Nowak RA (2009). Basigin-mediated gene expression changes in mouse uterine stromal cells during implantation. *Endocrinology*.

[5] Huet E, Gabison EE, Mourah S, Menashi S (2008). Role of emmprin/CD147 in tissue remodeling. *Connective Tissue Research*.

[6] Agrawal SM, Yong VW (2011). The many faces of EMMPRIN—roles in neuroinflammation. *Biochimica et Biophysica Acta*.

[7] Kanyenda LJ, Verdile G, Boulos S (2011). The dynamics of CD147 in Alzheimer's disease development and pathology. *Journal of Alzheimer's Disease*.

[8] Crosnier C, Bustamante LY, Bartholdson SJ (2011). Basigin is a receptor essential for erythrocyte invasion by Plasmodium falciparum. *Nature*.

[9] Kanekura T, Chen X (2010). CD147/basigin promotes progression of malignant melanoma and other cancers. *Journal of Dermatological Science*.

[10] Liu F, Cui L, Zhang Y (2010). Expression of HAb18G is associated with tumor progression and prognosis of breast carcinoma. *Breast Cancer Research and Treatment*.

[11] Xu J, Xu HY, Zhang Q (2007). HAb18G/CD147 functions in invasion and metastasis of hepatocellular carcinoma. *Molecular Cancer Research*.

[12] Li Y, Xu J, Chen L (2009). HAb18G (CD147), a cancer-associated biomarker and its role in cancer detection. *Histopathology*.

[13] Muramatsu T (2012). Basigin: a multifunctional membrane protein with an emerging role in infections by malaria parasites. *Expert Opinion on Therapeutic Targets*.

[14] Weidle UH, Scheuer W, Eggle D, Klostermann S, Stockinger H (2010). Cancer-related issues of CD147. *Cancer Genomics and Proteomics*.

[15] Xu J, Shen ZY, Chen XG (2007). A randomized controlled trial of licartin for preventing hepatoma recurrence after liver transplantation. *Hepatology*.

[16] Chen ZN, Mi L, Xu J (2006). Targeting radioimmunotherapy of hepatocellular carcinoma with iodine (131I) metuximab injection: clinical phase I/II trials. *International Journal of Radiation Oncology, Biology, Physics*.

[17] Ku XM, Liao CG, Li Y (2007). Epitope mapping of series of monoclonal antibodies against the hepatocellular carcinoma-associated antigen HAb18G/CD147. *Scandinavian Journal of Immunology*.

[18] Yu XL, Hu T, Du JM (2008). Crystal structure of HAb18G/CD147: implications for immunoglobulin superfamily homophilic adhesion. *Journal of Biological Chemistry*.

[19] Huang J, Ru B, Dai P, Cai W, Hong H (2012). Prediction of protein interaction sites using mimotope analysis. *Protein-Protein Interactions—Computational and Experimental Tools*.

[20] Huang J, Ru B, Dai P (2011). Bioinformatics resources and tools for phage display. *Molecules*.

[21] Huang J, Ru B, Zhu P (2012). MimoDB 2.0: a mimotope database and beyond. *Nucleic Acids Research*.

[22] Ru B, Huang J, Dai P (2010). MimoDB: a new repository for mimotope data derived from phage display technology. *Molecules*.

[23] Huang J, Ru B, Li S, Lin H, Guo FB (2010). SAROTUP: scanner and reporter of target-unrelated peptides. *Journal of Biomedicine and Biotechnology*.

[24] Negi SS, Braun W (2009). Automated detection of conformational epitopes using phage display peptide sequences. *Bioinformatics and Biology Insights*.

[25] Chen Z, Xing J, Zhang S Variable region gene of heavy/light chain of anti-human hepatoma monoclonal antibody HAb 18 and use thereof.

[26] Sircar A, Kim ET, Gray JJ (2009). RosettaAntibody: antibody variable region homology modeling server. *Nucleic Acids Research*.

[27] Tovchigrechko A, Vakser IA (2006). GRAMM-X public web server for protein-protein docking. *Nucleic Acids Research*.

[28] Wiehe K, Pierce B, Tong WW, Hwang H, Mintseris J, Weng Z (2007). The performance of ZDOCK and ZRANK in rounds 6-11 of CAPRI. *Proteins*.

[29] Wiehe K, Pierce B, Mintseris J (2005). ZDOCK and RDOCK performance in CAPRI rounds 3, 4, and 5. *Proteins*.

[30] Krissinel E, Henrick K (2007). Inference of macromolecular assemblies from crystalline state. *Journal of Molecular Biology*.

[31] Carter DM, Miousse IR, Gagnon JN (2006). Interactions between TonB from Escherichia coli and the periplasmic protein FhuD. *Journal of Biological Chemistry*.

[32] Derda R, Tang SKY, Li SC, Ng S, Matochko W, Jafari MR (2011). Diversity of phage-displayed libraries of peptides during panning and amplification. *Molecules*.

[33] Chen W, Guo WW, Huang Y, Ma Z (2012). PepMapper: a collaborative web tool for mapping epitopes from affinity-selected peptides. *PLoS One*.

[34] Chen WH, Sun PP, Lu Y, Guo WW, Huang YX, Ma ZQ (2011). MimoPro: a more efficient Web-based tool for epitope prediction using phage display libraries. *BMC Bioinformatics*.

[35] Huang YX, Bao YL, Guo SY, Wang Y, Zhou CG, Li YX (2008). Pep-3D-Search: a method for B-cell epitope prediction based on mimotope analysis. *BMC Bioinformatics*.

[36] Saphire EO, Parren PWHI, Pantophlet R (2001). Crystal structure of a neutralizing human IgG against HIV-1: a template for vaccine design. *Science*.

[37] Zhou T, Xu L, Dey B (2007). Structural definition of a conserved neutralization epitope on HIV-1 gp120. *Nature*.

[38] Belton RJ, Chen L, Mesquita FS, Nowak RA (2008). Basigin-2 is a cell surface receptor for soluble basigin ligand. *Journal of Biological Chemistry*.

[39] Cui HY, Guo T, Wang SJ (2012). Dimerization is essential for HAb18G/CD147 promoting tumor invasion via MAPK pathway. *Biochemical and Biophysical Research Communications*.

